# Globally Accurate Gaussian Process Potential Energy Surface and Quantum Dynamics Studies on the Li(^2^S) + Na_2_ → LiNa + Na Reaction at Low Collision Energies

**DOI:** 10.3390/molecules28072938

**Published:** 2023-03-24

**Authors:** Zijiang Yang, Hanghang Chen, Bayaer Buren, Maodu Chen

**Affiliations:** 1 Key Laboratory of Materials Modification by Laser, Electron, and Ion Beams (Ministry of Education), School of Physics, Dalian University of Technology, Dalian 116024, China; 2 School of Science, Shenyang University of Technology, Shenyang 110870, China

**Keywords:** potential energy surface, Gaussian process, ab initio, reaction dynamics, time-dependent wave packet

## Abstract

The LiNa_2_ reactive system has recently received great attention in the experimental study of ultracold chemical reactions, but the corresponding theoretical calculations have not been carried out. Here, we report the first globally accurate ground-state LiNa_2_ potential energy surface (PES) using a Gaussian process model based on only 1776 actively selected high-level ab initio training points. The constructed PES had high precision and strong generalization capability. On the new PES, the quantum dynamics calculations on the Li(^2^S) + Na_2_(*v* = 0, *j* = 0) → LiNa + Na reaction were carried out in the 0.001–0.01 eV collision energy range using an improved time-dependent wave packet method. The calculated results indicate that this reaction is dominated by a complex-forming mechanism at low collision energies. The presented dynamics data provide guidance for experimental research, and the newly constructed PES could be further used for ultracold reaction dynamics calculations on this reactive system.

## 1. Introduction

In recent years, preparing cold and ultracold molecules have received increasingly intensive attention owing to their important applications in quantum simulation, quantum many-body physics, quantum information processing and other cutting-edge fields [1,2,3,4,5,6,7,8,9,10,11,12]. In the ultracold regime, the reactive collisions between molecules are mainly controlled by quantum effects such as resonances, tunneling and quantum interference, since the translational de Broglie wavelength is much larger than the range of interaction potential. Therefore, studying ultracold reaction dynamics is of great significance for understanding hidden quantum mechanical behaviors and accurately detecting the microscopic mechanisms in chemical reaction processes.

Up to now, most of the experimental studies of ultracold chemical reactions have focused on interaction systems comprised of alkali metal dimers. In 2010, it was first confirmed that chemical reactions can proceed at ultracold conditions by measuring the loss of reactant molecules in the KRb + KRb → K_2_ + Rb_2_ reaction [13]. Hu et al. directly observed the K_2_Rb_2_ intermediates of the reaction between KRb molecules by preparing the reactant in the lowest rovibrational state at ultralow temperatures to restrict the number of accessible exit channels [14], showing that the lifetime of the intermediate is extended to several microseconds. Ye et al. studied in detail [15] the inelastic loss of optically trapped ultracold NaRb gases without and with the NaRb + NaRb → Na_2_ + Rb_2_ reaction. They found extremely similar loss and heating, regardless of the reactivities, and the collisions were outside the Wigner region although the temperatures of the molecules were sub-microkelvin. With the rapid development of experimental technologies, collision systems, such as RbCs + RbCs [16,17], NaK + NaK [18,19] and LiNa + LiNa [20] were also widely studied. Inspired by excellent experimental works, a variety of theoretical studies on these reactive systems have been carried out. Theoretical calculations of reaction dynamics not only provide guidance for the implementation of experimental research but also describe fully how the reaction takes place by providing details of the reaction process. The most accurate method for studying the microscopic mechanisms of a chemical reaction is to carry out rigorous quantum dynamics calculations on a high-quality potential energy surface (PES). The global full-dimensional PESs of K_2_Rb_2_ [21,22], Na_2_Rb_2_ [23] and Na_2_K_2_ [24] reactive systems have been constructed by combining high-level ab initio calculations and machine learning technologies, but the corresponding quantum dynamics calculations have not been reported, since performing full-dimensional quantum dynamics simulations for a four-atom reaction system dominated by a deep well requires huge computational cost, even at room temperature.

The reactive or non-reactive collisions between alkali metal atoms and heteronuclear alkali metal dimers, such as Na + LiNa [7], Na + NaK [19], K + NaK [9], and Rb + KRb [25] have also been studies recently. In theory, the current models and methods can implement rigorous quantum dynamics treatment on such triatomic reactive systems [26,27,28,29,30]. Among these reactions, the LiNa_2_ system has relatively fewer electron; thus, it is easier and more accurate to perform the electronic structure calculations. It is also an ideal candidate for studying the ultracold chemical reactions controlled by outside factors. In 2022, Son et al. demonstrated the magnetic control of reactive collision in an ultracold mixture of Na and NaLi [31]. They found that this system has a very small loss probability near short range, and the loss rate can be modified by a factor of 100 by controlling the phase of the scattering wave function by Feshbach resonance. Theoretical studies of this important reactive system have not been reported up to now. It is well known that the premise of accurately describing reaction dynamics behavior is to represent a globally high-precision PES. In this work, we constructed the first ground-state LiNa_2_ PES by combining high-level ab initio calculations and machine learning algorithms. In addition, previous studies concentrated on the reactions of alkali metal atoms with heteronuclear alkali metal dimers, whereas it is still a challenge to experimentally study the chemical reactions of alkali metal atoms with homonuclear alkali metal dimers at low temperatures, thus the dynamics characteristics of these type reactive systems have not been understood completely. We performed quantum dynamics calculations for the Li(^2^S) + Na_2_(*v* = 0, *j* = 0) → LiNa + Na reaction at the collision energy range of 0.001–0.01 eV on the newly constructed PES.

This paper is organized as follows. In Section 2, the computational details, methodology, and characteristics of the ground-state LiNa_2_ PES are described. In Section 3, the modified quantum time-dependent wave packet (TDWP) method is briefly introduced, and the quantum dynamics results, including reaction probabilities, the state-resolved scattering sections and the angular distributions of the product molecule are analyzed in detail. The conclusions of this work are presented in Section 4.

## 2. Ground-State LiNa_2_ PES

### 2.1. Ab Initio Calculations

The potential energies of ground-state LiNa_2_ (1^2^A′) are calculated within *C_s_* symmetry, as implemented in the Molpro 2012 package [32]. First, the Hartree-Fock method is applied to generate the single-configuration wavefunctions, and then the molecular orbitals are optimized by the complete active space self-consistent field (CASSCF) [33,34] calculations for the equally weighted three electronic states (1^2^A′, 2^2^A′ and 1^2^A″), and twelve (9a′ + 3a″) active orbitals are used for the three correlated valence electrons of the LiNa_2_ system. Finally, internally contracted multi-reference configuration interaction (icMRCI) [35,36] calculations including the Davidson correction (+Q) are carried out to correct the higher order correction energies using the CASSCF wavefunctions as reference. The cc-pwCVQZ [37] and def2-QZVPP [38] basis sets are adopted for Li and Na atoms, respectively. The reactant Jacobi coordinate (*r*, *R*, *θ*) is used to select molecular configurations, where *r* is the bond length of the two Na atoms, *R* represents the distance from the Li atom to the center of mass of the Na_2_ molecule, and *θ* is the angle between *R* and *r*. Ab initio calculations are performed in a wide configuration space, defined as 3.0 *a*_0_ ≤ *r* ≤ 30.0 *a*_0_, 0.0 *a*_0_ ≤ *R* ≤ 45.0 *a*_0_, 0° ≤ *θ*/degree ≤ 90°.

### 2.2. Actively Selecting Configurations and Fitting of PES

The rapid development of electronic structure calculation methods and continuous improvement of computational performance have enabled extremely high-precision ab initio energies for simple reactive systems. The main challenge in constructing multi-dimensional PESs is to represent the function between the potential energies and the molecular nuclear coordinates based on the discrete ab initio data. Fitting PESs with machine learning models has been gaining popularity in recent years, and using an artificial neural network (NN) [39,40,41,42,43,44,45,46,47,48,49,50,51,52,53,54] or a Gaussian process (GP) [55,56,57,58,59,60,61,62,63,64,65,66,67,68,69,70] are the two most common approaches. GP is a kernel-based supervised statistical learning method [71], which has been widely used to solve physical chemistry problems such as mapping high-dimensional PESs and simulating quantum scattering dynamics. The GP model offers two main advantages for constructing PESs compared with the other methods. One is that GP requires much fewer training data points than NNs, and offers a direct active learning scheme by providing the uncertainty of any molecular configurations; thus, the corresponding cost of ab initio calculations can be greatly reduced for multi-dimensional molecular systems. Another advantage is that no additional work is required to avoid overfitting behavior since the PESs of the relatively simple reactive systems can be represented by GP with simple kernels. A key drawback is that the numerical cost of training and evaluating a GP PES increases with the number of training data *n*, scaling as *O*(*n*^3^) and *O*(*n*), respectively. It is therefore necessary to construct PESs with the GP method based on as few points as possible. Here, the active selection of configurations and the construction of the ground-state LiNa_2_ PES are implemented by the GP model, and we briefly give the relevant theory and equations.

The inputs ***X*** = [***x***_1_,…, ***x****_n_*] of the GP model are the variables describing the interatomic distance of LiNa_2_. Permutational symmetry is an essential and important property of molecular systems. When the reactive system includes identical atoms, the resulting PES needs to be invariant with respect to permutations of like atoms. To ensure the permutation symmetry of the resulting PES, the permutation invariant polynomials (PIPs) [72,73] are used to treat the three bond lengths. The basic idea of PIP is to use coordinates with permutation symmetry to describe molecular configurations. For the AB_2_ type system, the three internuclear distances are transformed to the Morse-like variables, written as:

(1) $$p_{k}=\exp(-\alpha R_{k})\;(k=1,2,3)$$

where *α* is a range parameter, and here set as 0.2. The final symmetrized polynomial vector ***G*** = {*G_i_*} is structured as:

(2) $$G_{1}=(p_{1}+p_{3})/2$$

(3) $$G_{2}=p_{1}\times p_{3}$$

(4) $$G_{3}=P_{2}$$

The *n* observations ***y*** = [*y*_1_,…, *y_n_*] are the corresponding normalized potential energies. The vector-matrix form of the joint multivariate Gaussian distribution can be expressed as:

(5) y∼N(0,K(X,X))

where the mean function is set as zero, and ***K***(***X***, ***X***) is the covariance matrix with the elements of kernel function *k*(***x****_i_*, ***x****_j_*) that represents the similarity between ***x****_i_* and ***x****_j_*. Here, the anisotropic Matérn kernel with *v* = 2.5 is used as the covariance function, expressed as:

(6) $$k(x_{i},x_{j})=(1+\sqrt{5}\frac{d(x_{i},x_{j})}{l}+\frac{5}{3}\frac{d{\left(x_{i},x_{j}\right)}^{2}}{l^{2}})\exp(-\sqrt{5}\frac{d(x_{i},x_{j})}{l})+\delta_{ij}\sigma_{n}$$

where *d*(***x****_i_*, ***x****_j_*) is the Euclidean distance between ***x****_i_* and ***x****_j_*, and ***l*** = [*l*_1_, *l*_2_, *l*_3_] represents the length-scale vector. To reduce the risk of the ill covariance matrix, the noise terms *δ_ij_σ_n_* is added to the diagonal of ***K***. *σ_n_* and ***l*** form the hyperparameters that need to be optimized, denoted by ***θ***. The parameters of the kernel function can be determined by maximizing the following marginal likelihood:

(7) $$\log p(y|X,\theta )=-\frac{1}{2}y^{T}K^{-1}y-\frac{1}{2}\log\left|K\right|-\frac{n}{2}\log(2\pi )$$

According to the property of GP, a new point (x*, y*) that is not included in the training database, also follows this prior distribution:

(8) $$\left[\begin{array}{c} y\\ y^{*} \end{array}\right]∼N\left[0,\;\begin{array}{cc} K(X,X) & K^{*T}(x^{*},X)\\ K^{*}(X,x^{*}) & K^{*}{}^{*}(x^{*},x^{*}) \end{array}\right]$$

where K** = *k*(x*, x*), and K* vector consists of the covariance between x* and all the training data. The predicted mean of y* is:

(9) $$\mu (x^{*})=[K^{*}K+\sigma_{n}^{2}I{]}^{-1}y$$

and its variance is calculated by:

(10) $$\sigma^{2}(x^{*})=K^{**}-K^{*T}{\left[K+\sigma_{n}^{2}I\right]}^{-1}K^{*}$$

It can be seen from Equation (5) that predicting the potential energy of an unknown configuration by a GP PES requires the covariance matrices between this new geometry and all of the training data, and the product of the *n*-dimensional vector-vector is required to calculate. Therefore, to evolute a GP PES becomes difficult with an increase of training data, and the subsequent dynamics calculations are limited significantly. Therefore, for precision, it is crucial to effectively reduce the number of training points for constructing a reactive PES with the GP model. Here, two active learning schemes, namely the highest variance search and highest absolute error search, are compared to automatically select the training data in the process of representing the global ground-state LiNa_2_ PES. We sample 30 molecular configurations using the Latin hypercube approach [74] and calculate the corresponding energies as the initial database, and a total of 13,453 ab initio points that cover the entire coordination space are selected as the test database. The test database serves two purposes: providing the candidate data to be added to the training database and testing the generalization performance of the resulting GP PES. First, a rough GP PES is structured using the initial database, and the predictive variances and absolute errors of all the test data points are calculated by this initial PES. The point with the highest variance or absolute error is removed from the test database and added to the training database. The training set is circularly updated until the highest absolute error converges to the set energy value, and the final GP PES is determined simultaneously.

**Figure 1:**
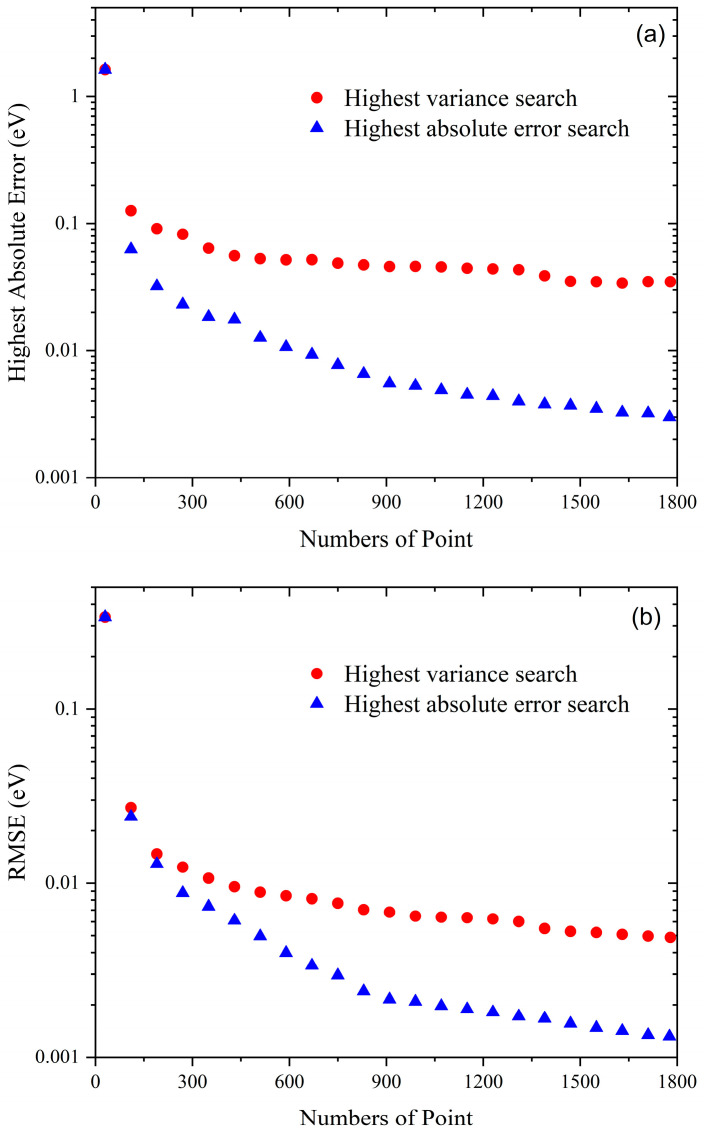
Highest absolute error (**a**) and RMSE (**b**) of the remaining test data as a function of the number of training points for the LiNa_2_ PES modeled by the GP model based on the two active learning schemes.

Figure 1a,b shows the highest absolute error and root mean square error (RMSE) of the remaining test data calculated by the GP model based on the two active learning strategies as a function of the number of training points. The highest absolute error and RMSE calculated by the GP PES with 30 points are 1.63 eV and 0.34 eV, respectively, presenting very poor generalization performance. For both the two active schemes, the highest absolute error and RMSE decrease with the increase of the number of training data, but the rate of decline of the highest absolute error search method obviously outperforms the highest variance search method. A previous study in constructing the intermolecular PESs of CO_2_–Ne, CO_2_–H_2_, and Ar_3_ systems also shows a similar variation tendency [63]. The RMES and highest absolute error of the remaining test data reach meV magnitude when the number of training points increases to 421 and 609, respectively, for the highest absolute error search method. The convergency value of the highest absolute error is set as 3 meV, and 1746 training points are actively selected. Therefore, the final LiNa_2_ PES is represented using the GP model by 1776 ab initio points, and the corresponding RMES of the remaining test data is 1.32 meV.

**Figure 2:**
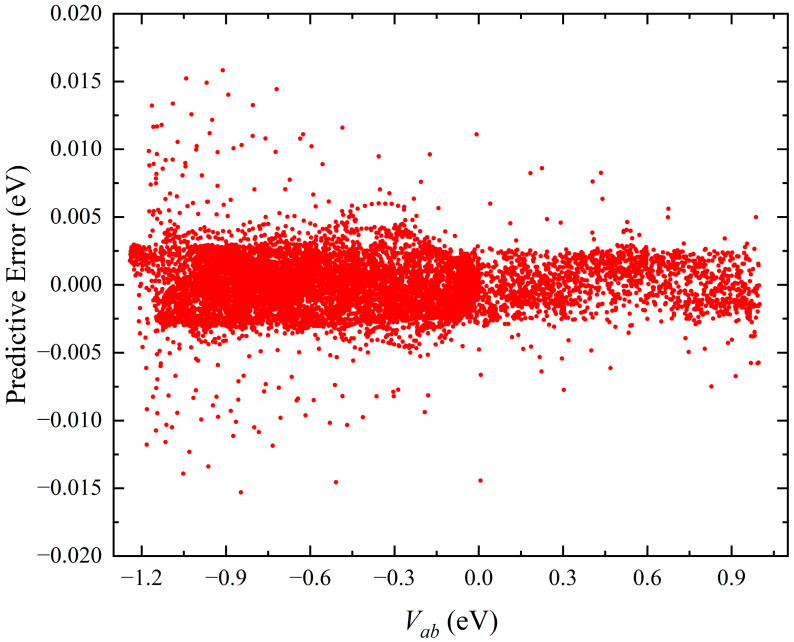
Predictive error distributions in the test database (13,453 points) of the ground-state LiNa_2_ PES constructed by the GP model with 1776 points.

Figure 2 shows the distribution of the predictive errors for all the test data points, which are defined by the difference between the energy values obtained on the GPR PES and the original ab initio data. The potential energy values are relative to the dissociation limit of Li–Na–Na. In the whole energy range, the predictive errors maintain very small values, suggesting that the GPR PES can accurately reproduce the high-level ab initio energies for all the test configurations. The predictive RMES for the test database is only 1.85 meV, and the highest absolute error is 15.82 meV. The proportion of points with an absolutely predictive error of less than 5 meV can reach 98.6% of all the test configurations, implying that the constructed GP PES is globally accurate and has excellent generalization performance.

### 2.3. Topographic Features of PES

**Figure 3:**
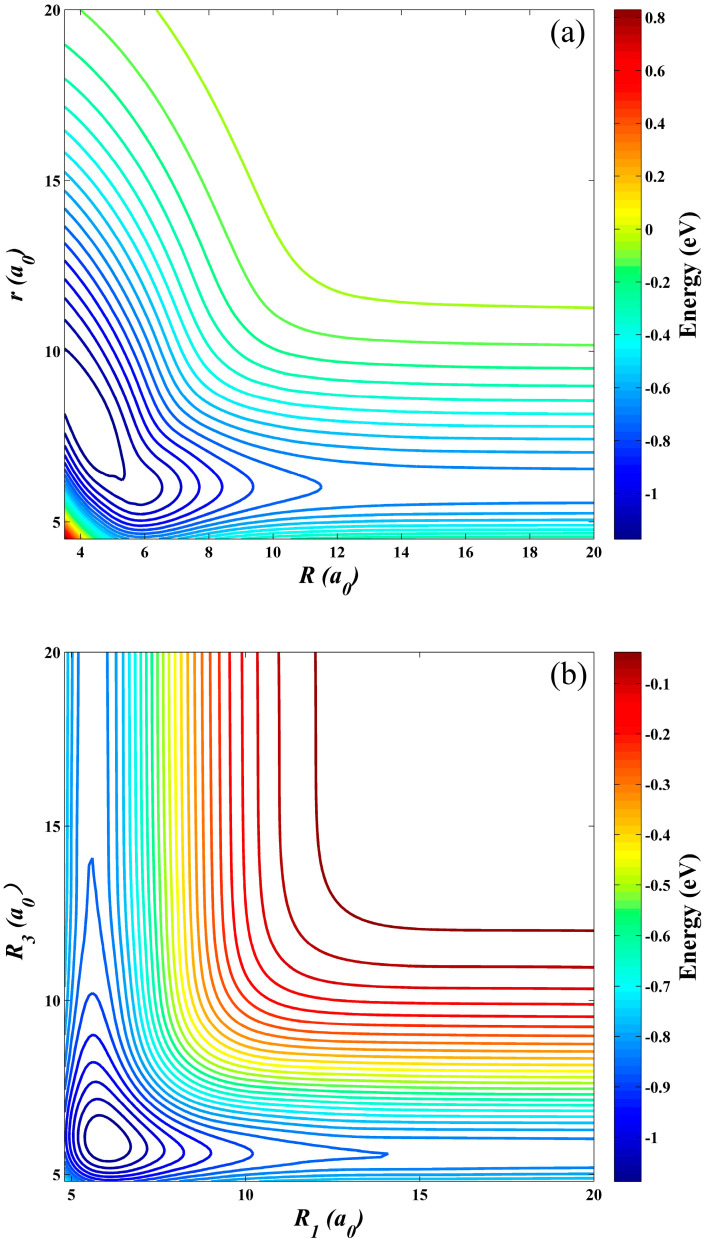
Contour plots of the ground-state LiNa_2_ PES at *C_2v_* (**a**) and *D_∞h_* (**b**) symmetries.

Figure 3a,b displays contour plots of the ground-state LiNa_2_ PES at *C_2v_* and *D_∞h_* symmetries, respectively. For Figure 3a, there is a well with the lowest energy value of −1.24 eV at *r* = 7.78 *a*_0_, *R* = 4.78 *a*_0_, and it is also the equilibrium geometry of the ground-state LiNa_2_ system. At low temperatures or ultracold conditions, the Li atom slowly moves along the midperpendicular of the Na_2_ molecule with the remarkable elongation of the Na–Na bond, and a Li–Na–Na complex is formed before the Na-Na bond is broken. Figure 3b shows the excellent exchange symmetry of the constructed PES about the two same Na atoms. There is a well with the depth of 1.12 eV at *R*_1_ = *R*_3_ = 5.92 *a*_0_. It is worth mentioning that no saddle point or cusp caused by the conical intersection of the first excited state is presented for the two special symmetries, suggesting that electronic non-adiabatic coupling has little effect on this reactive system.

**Figure 4:**
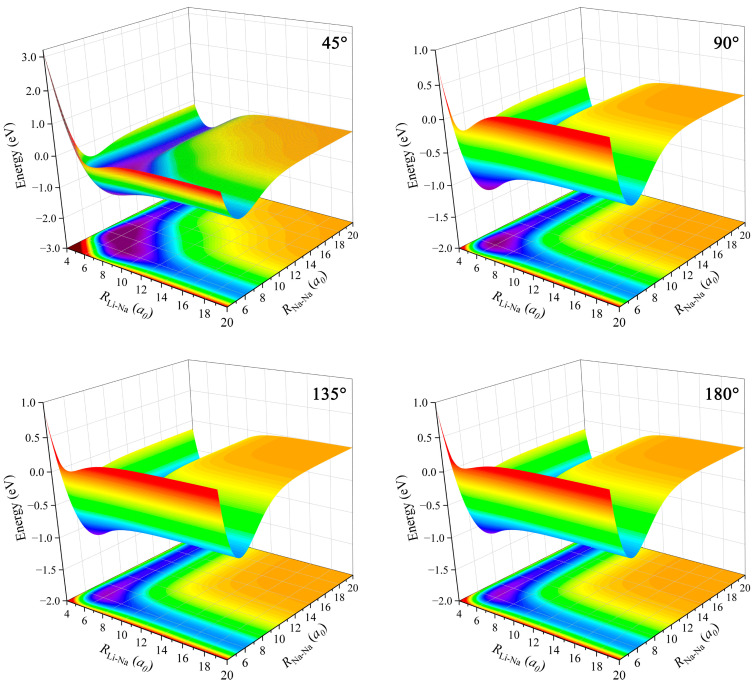
Three-dimensional diagrams and the corresponding contour maps of the ground-state LiNa_2_ PES at fixed Li–Na–Na angles (45°, 90°, 135° and 180°).

Three-dimensional diagrams and the corresponding contour maps of the ground-state LiNa_2_ PES at fixed Li–Na–Na angles (45°, 90°, 135° and 180°) are shown in Figure 4. The resulting PES modeled by the GP model is very smooth in the whole coordinate space, and no non-physical well and barrier occur at large Li–Na or Na–Na bond length for each approaching angle. There is a valley at the left and a valley at the right of the LiNa_2_ PES with the fixed Li–Na–Na angle, which correspond to the Na(^2^S) + LiNa channel and Li(^2^S) + Na_2_ channel, respectively, and the two channels are connected by a potential well. The energy value of the left valley is lower than the right valley, implying that the Li(^2^S) + Na_2_ → LiNa + Na reaction is exothermic. It can be seen that the topographic features of the LiNa_2_ PES have relatively small changes with the increase of Li–Na–Na angle, especially for large approaching angles; thus, the GP model is suitable for the construction of the globally accurate PES for the LiNa_2_ system.

**Figure 5:**
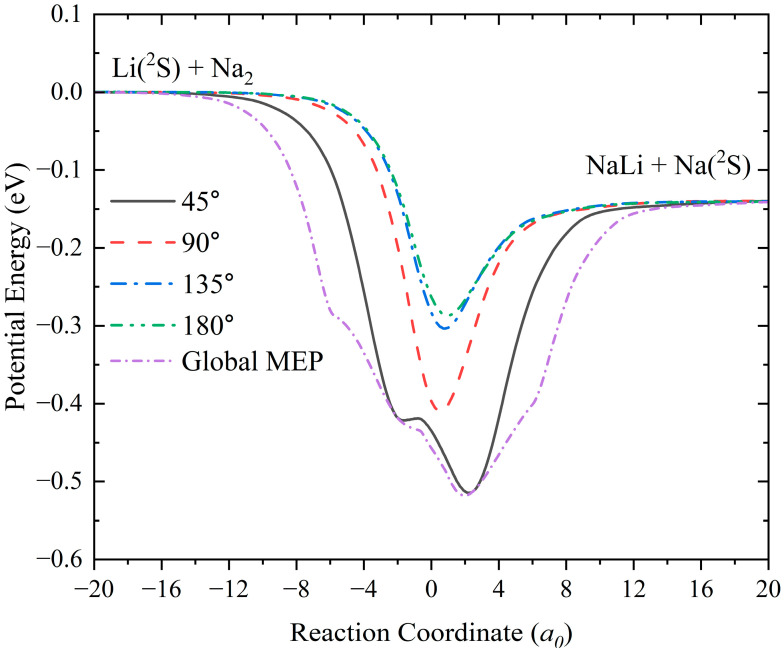
MEPs at four Li–Na–Na angles (45°, 90°,135°, and 180°) and global MEP of the Li(^2^S) + Na_2_ → LiNa + Na reaction calculated by the GP PES.

To more intuitively present the characteristics of the reactive process, Figure 5 shows the minimum energy paths (MEPs) of the title reaction at different Li–Na–Na angles (45°, 90°, 135° and 180°) calculated by the GP PES and the global MEP obtained by scanning the LiNa_2_ PES to obtain the minimal energy in the reaction coordinate. The reactive path is dominated by a well for each approaching angle, and the depth of the well gradually becomes shallower with the increase of the Li–Na–Na angle, indicating that the forming complex has a shorter lifetime when the Li atom collides with the Na_2_ molecule with a large approaching angle. The global MEP also features a potential well with a depth of 0.52 eV, corresponding to the geometry of the global minimum energy shown in Figure 3a. There is no barrier for each reactive path, thus the title reaction can proceed at ultracold temperatures by any collision approach. At low collision energy or temperature, the reaction occurs along the global MEP, and the other paths are gradually opened with the increase of collision energy. The exothermicity of the title reaction determined on the GP PES is 0.14 eV.

**Figure 6:**
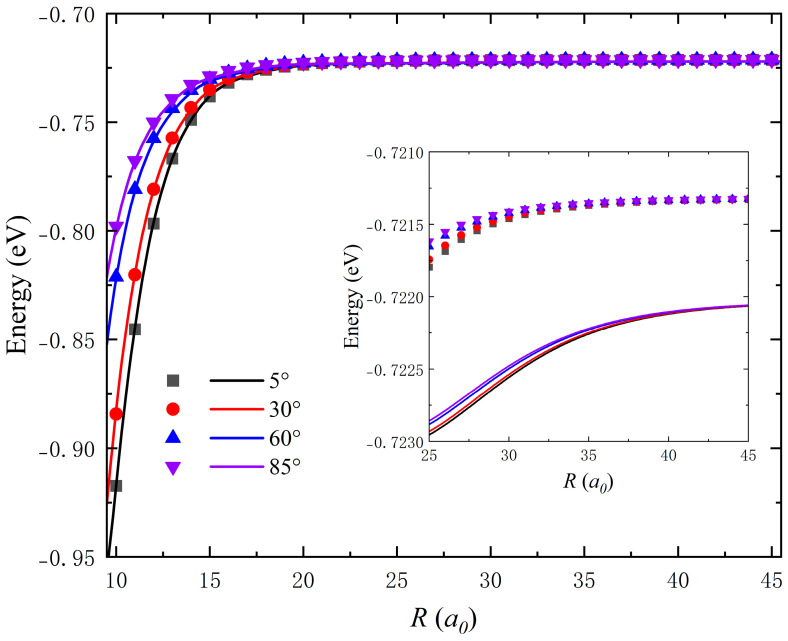
Long-range interaction potentials obtained on the GP PES along the radial coordinate at four fixed Jacobi angles (*θ* = 5°, 30°, 60° and 85°) with the bond length of Na_2_ fixed at 6.01 *a*_0_, in comparison with the original ab initio energies.

For the ultracold reaction, long-range interaction in the reactant channel is crucial for the dynamics calculations, and the minor change of the long-range potential may generate distinct dynamics characteristics. Figure 6 displays the long-range interaction potentials along the radial coordinate obtained on the GP PES at four fixed Jacobi angles (*θ* = 5°, 30°, 60° and 85°) with the bond length of Na_2_ fixed at 6.01 *a*_0_. It is clear that the fitted long-range potentials are in good agreement with the original high-level ab initio values for each Jacobi angle, and the corresponding difference is less than 1 meV. There is still minor interaction at the long-range region until *R* reaches around 40 *a*_0_, and the GP PES can reproduce this variation tendency well. Therefore, the GPR PES is sufficiently accurate for describing the long-range interaction. According to the above results and analysis, it can be concluded that the constructed ground-state LiNa_2_ PES is globally accurate, which is suitable for the dynamics calculations of the Li(^2^S) + Na_2_ reactive system under low temperatures even ultracold conditions.

## 3. Quantum Dynamics Calculations

Quantum mechanical approaches to calculating molecular collisions and reaction dynamics can be divided by the time-independent close coupling (TICC) method and TDWP method. The cost of the numerical evolution of the TICC grows cubically as a function of the number of channels, so it is very challenging to apply this method to reactive systems with a large number of channels due to the steep scaling laws. Compared with the TICC method, the TDWP method features much better expansibility, which is suitable for almost all triatomic reactive systems. However, the TDWP method has significant difficulty in treating ultracold scattering, which is because the large de Broglie wavelengths associated with ultracold reactions require an extremely large range of grids in the scattering coordinates, and the propagation time is very long owing to the slow movement of the wave packet. We developed recently a grid-based TDWP method [29], which can be used to accurately calculate the adiabatic and non-adiabatic dynamics of triatomic reactive systems. The main improvement of this method is to divide the total scattering wavefunction into long-range asymptotic and the interaction regions, and different set basis functions are selected in different parts. This new method has been used in the dynamics calculations of several typical systems, such as the H + LiH^+^ [75], H + BeH^+^ [76] and H + NaH [77] reactions. More details about the improved TDWP are provided in [28,29,30], and here only some important equations are listed.

The Hamiltonian of the Li(^2^S) + Na_2_ reactive system in the body-fixed representation can be expressed as:

(11) $$\hat{H}=-\frac{\hbar^{2}}{2\mu_{R}}\frac{\partial^{2}}{\partial R_{}^{2}}-\frac{\hbar^{2}}{2\mu_{r_{}}}\frac{\partial^{2}}{\partial r_{}^{2}}+\frac{{\left(\hat{J}-\hat{j}\right)}^{2}}{2\mu_{R}R_{}^{2}}+\frac{{\hat{j}}^{2}}{2\mu_{r_{}}r_{}^{2}}+\hat{V}$$

where *µ_R_* and *µ_r_* represent the corresponding reduced masses associated with *R* and *r* coordinates, *J* is the total angular momentum quantum number of LiNa_2_, and *j* is the rotational angular momentum quantum number of the Na_2_ molecule. *V* is the potential energy obtained on the newly constructed GP PES. The total wavefunction can be expressed as:

(12) $$\Psi^{IJM_{\varepsilon }}(R,r)=\sum_{K}D_{MK}^{J_{\varepsilon }}(\Omega )\psi^{IJ_{\varepsilon }}(R,r,\theta ;K)$$

where *K* is the projection of *J* on the *z*-axis of the body-fixed frame in the Li(^2^S) + Na_2_ channel, $D_{MK}^{J_{\varepsilon }}$ is the normalized Wigner rotation matrix, and *Ω* denotes the Euler angle. In this work, the reactant coordinate-based method [78,79] is used to extract the scattering matrix, and the evaluation of the wave packet is implemented by the split operator method [80]. The state-to-state reaction probability calculated by the scattering matrix is expressed as:

(13) $$P_{vj\leftarrow v_{0}j_{0}}^{J}=\frac{1}{2j_{0}+1}\sum_{K}{\sum_{K_{0}}\left|S_{vjK\leftarrow v_{0}j_{0}K_{0}}^{J}\right|}^{2}$$

The state-resolved ICSs calculated by summing the reaction probabilities of all *J* values:

(14) $$\sigma_{vj\leftarrow v_{0}j_{0}}=\frac{\pi }{(2j_{0}+1)k_{v_{0}j_{0}}^{2}}\sum_{K}\sum_{K_{0}}\sum_{J}\left(2J+1\right){\left|S_{vjK\leftarrow v_{0}j_{0}K_{0}}^{J}\right|}^{2}$$

where $k_{v_{0}j_{0}}$ is the momenta in the entrance channel. The state-resolved differential cross sections (DCSs) can be calculated by:

(15) $$\frac{d\sigma_{vj\leftarrow v_{0}j_{0}}(\vartheta ,E)}{d\Omega }=\frac{1}{\left(2j_{0}+1\right)}{\sum_{K}\sum_{K_{0}}\left|\frac{1}{2ik_{v_{0}j_{0}}}\sum_{J}\left(2J+1\right)d_{KK_{0}}^{J}(\vartheta )S_{vjK\leftarrow v_{0}j_{0}K_{0}}^{J}\right|}^{2}$$

where ϑ denotes the scattering angle of the product molecule, and $d_{KK_{0}}^{J}(\vartheta )$ is the element of the reduced Wigner rotation matrix.

Herein, the initial rovibrational state of the reactant Na_2_ molecule is set as *v*_0_ = 0, *j*_0_ = 0, and the number of partial waves is calculated up to *J* = 62, which can obtain convergent ICS and DCS up to 0.010 eV. The dynamics parameters of the TDWP calculations determined by numerous convergence tests are given in Table 1. To ensure the wave packet can accurately evolute to the low energy regions, an extremely large range of grid coordinates, a very long propagation time is used, and the initial wave packet is placed far away.

**Figure 7:**
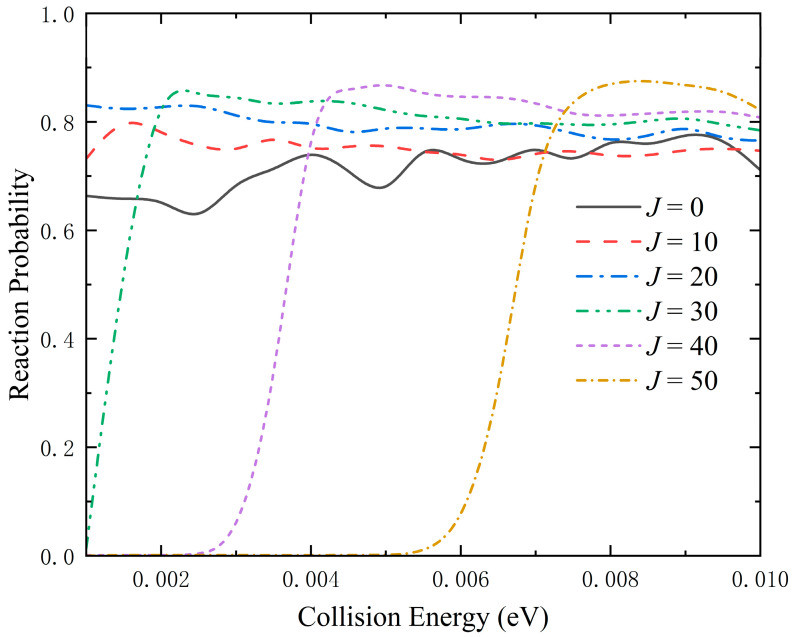
Collision energy dependence of total reaction probabilities with six partial waves (*J* = 0, 10, 20, 30, 40 and 50) of the Li(^2^S) + Na_2_(*v* = 0, *j* = 0) → LiNa + Na reaction calculated by the improved TDWP method on the GP PES.

The collision energy dependence of total reaction probabilities of the Li(^2^S) + Na_2_(*v*_0_ = 0, *j*_0_ = 0) → LiNa + Na reaction at six partial waves (*J* = 0, 10, 20, 30, 40 and 50) are presented in Figure 7. For the curve of *J* = 0, there is no reaction threshold due to the exothermicity and barrierless MEP. Furthermore, it exhibits relatively smooth oscillations, which is because the well on the global MEP can support bound and quasi-bound states, and the intermediate LiNa_2_ and quantum resonances are formed. With the increase of the *J* value, the reaction threshold appears, and the resonance structures gradually disappear since the increasing centrifugal potential barrier reduces the depth of the effective potential well. The increase of threshold and the convergence of reaction probabilities are relatively slow because of the large, reduced mass of the Li + Na_2_ system.

**Figure 8:**
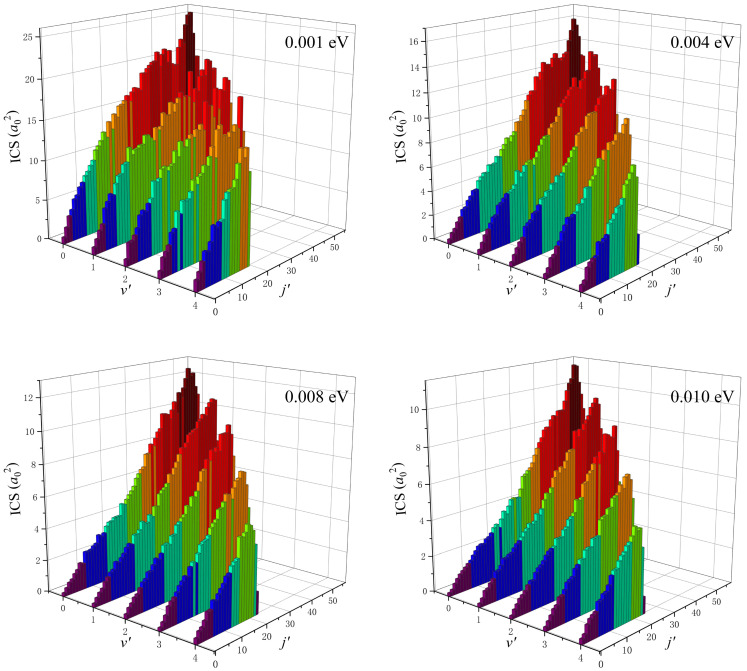
Rovibrationally state-resolved ICSs of the product LiNa molecules of the Li(^2^S) + Na_2_(*v*_0_ = 0, *j*_0_ = 0) → LiNa + Na reaction at four collision energies (0.001, 0.004, 0.008 and 0.010 eV) calculated by the improved TDWP method on the GP PES.

The rovibrationally state-resolved ICSs of the product LiNa molecules of the Li(^2^S) + Na_2_(*v*_0_ = 0, *j*_0_ = 0) → LiNa + Na reaction at four collision energies (0.001, 0.004, 0.008 and 0.010 eV) are shown in Figure 8. It is clear that the total ICS value decreases with the increase of collision energy, which is consistent with the features of the exothermic reaction without a barrier. For the collision energy of 0.001 eV, the product molecules can be vibrationally excited to *v*′ = 4, and the maximum rotational quantum number can reach *j*′ = 55 at the vibrational ground state. When the collision energy increases to 0.010 eV, no new vibrational channels are opened, and only two more rotational states are available compared to the 0.001 eV collision energy at *v*′ = 0. At the selected four collision energies, the distributions of rovibrational states of the product molecules are similar. There exists a population inversion of the rotational quantum number, whereas the vibrationally state-resolved ICS values monotonically decrease as the increase of *v*′, and, in addition, the product molecules prefer to populate at vibrationally cold and rotationally hot states. The characteristics of the distributions of rovibrational states suggest that the title reaction follows the complex-forming mechanism at low collision energies.

**Figure 9:**
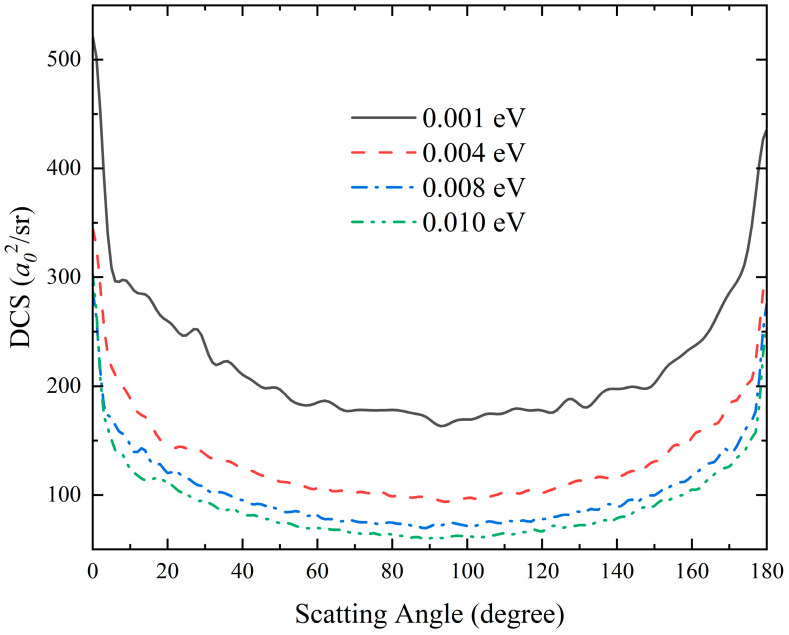
Total DCSs of the product LiNa molecules of the Li(^2^S) + Na_2_(*v* = 0, *j* = 0) → LiNa + Na reaction at four collision energies (0.001, 0.004, 0.008 and 0.010 eV) as a function of scattering angle calculated by the improved TDWP method on the GP PES.

To present the dynamics mechanisms more intuitively, based on the features of the angular distributions of the product molecules, Figure 9 presents the total DCSs of the product LiNa molecule of the Li(^2^S) + Na_2_(*v*_0_ = 0, *j*_0_ = 0) → LiNa + Na reaction varying with the scattering angle at four collision energies (0.001, 0.004, 0.008 and 0.010 eV). It can be seen that the peak values of the DCS curves are located at the two extreme angles (0° and 180°) for each collision energy, and the scattering angular distributions are symmetric with respect to 90°, showing apparent statistical behaviors. This is because the reaction almost completely proceeds along the global MEP at such low collision energies; thus, the complex LiNa_2_ is formed in the potential well of the reaction path, and there is enough time to evenly distribute the forward and backward scattering product molecules. The forward-backward symmetric DCSs further indicate that the complex-forming mechanism plays a dominant role in the range of studied collision energies.

## 4. Conclusions

In this paper, the first globally accurate ground-state LiNa_2_ PES is structured by combining the high-level ab initio calculations and the GP model. A total of 1776 training points calculated by the icMRCI +Q method were actively selected based on the highest absolute error to represent the GP PES, and the predictive REMS of 13,453 test points that cover the entire coordinate space by the constructed PES is only 1.85 meV, showing the high precision and strong generalization performance of the GP PES. There were multiple potential wells on the LiNa_2_ PES, and no saddle point and barrier were present. On the newly constructed PES, quantum dynamics calculations were performed on the Li(^2^S) + Na_2_(*v* = 0, *j* = 0) → LiNa + Na reaction in the 0.001–0.010 eV collision energy range using the improved TDWP method. The curves of reaction probabilities at low-order partial waves showed quantum resonance structures due to the existence of the well on the global MEP. The product LiNa molecules preferred to populate at low-vibrational and high-rotational states, and the total DCSs exhibited forward-backward symmetric angular distributions. The calculated dynamics results suggest that the title reaction follows the complex-forming mechanism in the range of selected collision energy.

## Figures and Tables

**Table 1:** Main numerical parameters in the TDWP calculations.

Li(^2^S) + Na_2_(*v* = 0, *j* = 0) → LiNa + Na	
*R* *r* Rotational basis Initial wave packet	*R* ∈ [0.1 *a*_0_, 100.0 *a*_0_], *N_R_* = 999, $N_{R}^{\mathrm{Int}}$ = 249 *r* ∈ [3.0 *a*_0_, 30.0 *a*_0_], *ν*_Int_ = 299, *ν*_Asy_ = 9 *j*_Int_ = 179, *j*_Asy_ = 69 *R_c_* = 50.0 *a*_0_, *δ* = 0.5 *a*_0_, *E_c_* = 0.04 eV
Propagation time Time step	2,000,000 a.u. for *J* ≤ 10 1,200,000 a.u. for *J* > 10 1,000,000 a.u. for *J* > 25 800,000 a.u. for *J* > 40 Δ*_t_* = 50 a.u.

## Data Availability

The data that support the findings of this study are available from the corresponding author upon reasonable request.
